# Molecular Evolution of Peste des Petits Ruminants Virus

**DOI:** 10.3201/eid2012.140684

**Published:** 2014-12

**Authors:** Murali Muniraju, Muhammad Munir, AravindhBabu R. Parthiban, Ashley C. Banyard, Jingyue Bao, Zhiliang Wang, Chrisostom Ayebazibwe, Gelagay Ayelet, Mehdi El Harrak, Mana Mahapatra, Geneviève Libeau, Carrie Batten, Satya Parida

**Affiliations:** The Pirbright Institute, Pirbright, UK (M. Muniraju, M. Munir, M. Mahapatra, C. Batten, S. Parida);; National Institute for Animal Biotechnology, Hyderabad, India (A.R. Parthiban, S Parida);; Animal Health and Veterinary Laboratories Agency, Weybridge, UK (A.C. Banyard);; China Animal Health and Epidemiology Centre, Qingdao, China (J. Bao, Z. Wang);; National Animal Disease Diagnostics and Epidemiology Centre, Entebbe, Uganda (C. Ayebazibwe);; National Veterinary Institute, Debre Zeit, Ethiopia (G. Ayelet);; Société de Productions Pharmaceutiques et Vétérinaires, Rabat, Morocco (M. El Harrak);; Le Centre de Cooperation Internationale en Recherche Agronomique pour le Développement, Montpellier (G. Libeau)

**Keywords:** peste des petits ruminants virus, PPRV, rinderpest virus, measles virus, viruses, emergence, Bayesian phylogenetics, phylogeography, nucleotide substitution rates, time to most recent common ancestor, TMRCA, divergence, evolution, selection pressure, small ruminants, wildlife

## Abstract

Despite safe and efficacious vaccines against peste des petits ruminants virus (PPRV), this virus has emerged as the cause of a highly contagious disease with serious economic consequences for small ruminant agriculture across Asia, the Middle East, and Africa. We used complete and partial genome sequences of all 4 lineages of the virus to investigate evolutionary and epidemiologic dynamics of PPRV. A Bayesian phylogenetic analysis of all PPRV lineages mapped the time to most recent common ancestor and initial divergence of PPRV to a lineage III isolate at the beginning of 20th century. A phylogeographic approach estimated the probability for root location of an ancestral PPRV and individual lineages as being Nigeria for PPRV, Senegal for lineage I, Nigeria/Ghana for lineage II, Sudan for lineage III, and India for lineage IV. Substitution rates are critical parameters for understanding virus evolution because restrictions in genetic variation can lead to lower adaptability and pathogenicity.

## Abstract 2

Sequence data will increase understanding of virus evolution, adaptability, and pathogenicity.

Peste des petits ruminants is a highly contagious and devastating viral disease of small ruminants that is endemic to much of Africa, the Middle East, and Asia (*1**,**2*). The causative agent, PPRV virus (PPRV), belongs to the family *Paramyxoviridae, *genus *Morbillivirus* (*3*) and groups with rinderpest virus (RPV), measles virus (MV), and canine distemper virus. Sheep and goats are the major hosts of PPRV, and infection has also been reported in a few wild small ruminant species (*2*). Researchers have speculated that RPV eradication has further enabled the spread of PPRV (*4**,**5*). Transmission of PPRV from infected goats to cattle has been recently reported (*6*), and PPRV antigen has been detected in lions (*7*) and camels (*8*). These reports suggest that PPRV can switch hosts and spread more readily in the absence of RPV (*4**,**6**,**8*). This host range switch had previously been seen after eradication of smallpox virus, which created a niche for monkeypox and cowpox viruses to cross the species barrier into humans (*4*).

PPRV has caused numerous serious epidemics in small ruminant populations across sub-Saharan Africa, the Middle East, and major parts of the Indian subcontinent where PPRV is considered endemic (*1*). In recent years, PPRV has extended its range southward in Africa as far as southern Tanzania (2008) and the Democratic Republic of Congo and Angola (2012). PPR outbreaks have also been reported across North Africa, including within Tunisia (2006), Morocco (2008), and Algeria (2011). In addition, within Europe, Turkey reported ≈20 laboratory-confirmed PPR outbreaks in sheep and goats during 2011–2012. In southwestern Asia, the virus spread to Tibet (2007) and has recently been reported throughout China (2013–2014). It is unclear what factors have favored emergence and spread of the disease, but millions of small ruminants across these regions must now be considered at high risk for infection with PPRV (*9*). The huge effect on small ruminant production has resulted in PPRV emerging as a global animal health concern.

The molecular epidemiology of PPRV, which is based on sequence comparison of a small region of the fusion (F) gene (322 nt) or the nucleoprotein (N) gene (255 nt), has identified 4 distinct lineages (I–IV) of PPRV (*2*). However, this analysis has not generated much information on the evolution and dispersal of PPRV lineages. Lineage I PPRV had gone undetected for 19 years being detected in Senegal in 1994. Lineage IV PPRV, which was believed initially to be confined to India and the Middle East, now has a wider geographic presence and appears to be evolving rapidly. Many aspects of PPRV evolution, such as ancestral virus location, divergence and time of origin, and historical and geographic patterns of spread, are poorly understood (*10*). A better understanding of the evolution of PPRV would enable prediction of how these viruses will lead to further outbreaks and epidemics and provide data for control strategies.

Advanced sequencing technologies have enabled molecular epidemiologic studies of viruses in which whole gene and complete genome data are used to enhance and clarify the evolutionary dynamics of viral infectious disease (*11*). We analyzed genome data for all 4 lineages of PPRV. This analysis will enable a more precise evolutionary and phylogenetic assessment of the relationships between lineages by reducing the associated estimation errors and increased higher confidence in estimates.

## Materials and Methods

### Complete Genome Sequencing of PPRV

Complete genome sequencing of 7 PPRV isolates (4 from lineage III and 3 from lineage IV) was performed according to the methods described by Muniraju et al. (*12*). Detailed information for each of the isolates is shown in Table 1.

**Table 1:** Peste des petits ruminants virus isolates used for complete genome analysis

Virus isolates	GenBank accession no.	Lineage	Source (reference)
Ivory Coast/1989	EU267273	I	Goat (*13*)
Nigeria/1976	EU267274	II	Sheep (*13*)
Nigeria/1975/1	X74443	II	Goat (14), vaccine strain
Uganda/2012*	KJ867543	III	Goat
UAE/1986*†	KJ867545	III	Dorcas gazelle (*15*)
Oman/1983*	KJ867544	III	Goat (*16*)
Ethiopia/1994*	KJ867540	III	Goat (*17*)
Ethiopia/2010*	KJ867541	IV	Goat
India/Sungri/1996*	KJ867542	IV	Goat (provided by Intervet International B.V, Boxmeer, the Netherlands), vaccine strain
Morocco/2008*	KC594074	IV	Goat (*12*)
China/Tibet Bharal/2008	JX217850	IV	Bharal, *Pseudois nayaur* (*18*)
China/Tibet33/2007	JF939201	IV	Goat (*19*)
China/TibetGeg30/2007	FJ905304	IV	Goat (*19*)
Turkey/2000	NC006383	IV	Sheep (*20*)

*Whole genome sequencing was conducted. †UAE, United Arab Emirates.

### Sequence Datasets

In addition to the 7 complete genomes sequences of PPRV generated in this study, another 7 complete genome sequences were obtained from GenBank (Table 1). However, of these 14 full genome sequences, Nigeria 1975/1 and Sungri 1996 represent vaccine strains generated after extensive serial passage of virus. Therefore, the evolutionary rate and time to most recent common ancestor (TMRCA) were compared with and without inclusion of vaccine strains. The complete genome sequences of 2 clinical isolates each from RPV (GenBank accession nos. AB547189 and X98291) and MV (accession nos. AF266288 and JF791787) and 12 PPRV isolates, excluding vaccine strains, (Table 1) were used for estimation of evolutionary rate and TMRCA. Furthermore, the coding and noncoding sequences of individual structural genes of PPRV (excluding vaccine strains) were used in this study.

Partial N gene sequences of PPRV (nucleotide positions 1253–1507) that have a detailed history of collection date and place were obtained from GenBank (available up to August 2013). These partial sequences were aligned by using the ClustalW algorithm in BioEdit software v7.2.0. (*21*) and edited to remove unreliable sequences/regions. Furthermore, the identical sequences originating from the same geographic location, host, and year were excluded to avoid redundancy in subsequent analysis. The final dataset (partial N gene) contained 159 sequences sampled over a period of 45 years (1968–2012).

### Selection Analysis

The nucleotide and amino acid sequence differences between the PPRV lineages for 12 complete genome sequences were estimated by using BioEdit software v7.2.0. Analyses of selection pressure in individual PPRV genes was performed by obtaining mean ratios of nonsynonymous (dN) to synonymous (dS) substitutions per site. The dN/dS was calculated by using codon-based maximum likelihood approaches with the single-likelihood ancestor method implemented in hypothesis testing using the phylogenies package (*22*) (http://www.datamonkey.org).

### Bayesian Time-Scaled Phylogenetic Analysis

Molecular evolutionary rate and divergence times were co-estimated. A Bayesian maximum clade credibility (MCC) phylogenetic tree was constructed by using Bayesian Markov chain Monte Carlo (MCMC) analysis and Bayesian evolutionary analysis sampling trees (BEAST) software package v1.8.0 (*23*), and BEAST runs were performed by using the CIPRES Science Gateway (*24*). For each sequence dataset, the best-fit nucleotide substitution model was determined on the basis of Akaike information criterion scores using JModel Test software v2.1.4 (*25*). An input file for BEAST analysis was obtained by using Bayesian evolutionary analysis utility software v1.8.0, in which sequences were tip dated according to the year of collection. Four molecular clock models (strict, uncorrelated lognormal distribution, uncorrelated exponential distribution [UCED], and random) were tested alongside different demographic models (nonparametric Bayesian skyline plot and the parametric constant and exponential growth), and the best models were selected by means of a Bayes factor (BF) test (*26*) using marginal likelihoods values (2lnBF>2) obtained from Tracer v1.5 software (http://beast.bio.ed.ac.uk/tracer).

For each analysis, 2 independent MCMC chains were run to get a final output of 10,000 trees (ESS >200 for all the parameters estimated) and were assessed for their proper mixing, convergence, and consistency by Tracer v1.5 with 10% burn in. The 2 individual runs were combined by using LogCombiner v1.8.0 in the BEAST software package. The nucleotide substitution rate (substitutions/site/year) and the TMRCA (year) values were obtained from Tracer v1.5. The posterior tree distributions were summarized by using TreeAnnotator (http://beast.bio.ed.ac.uk/treeannotator) and exclusion of the first 10% of the trees as burn in. Phylogenetic MCC tree with median node heights were visualized in FigTree software v1.4.0 (http://www.molecularevolution.org/software/phylogenetics/figtree). Furthermore, the demographic history of PPRV was studied by using partial N gene dataset and less restrictive Bayesian skyline plot (BSP) models in which the changing profile of genetic diversity is plotted against time.

### Phylogeographic Reconstruction

Bayesian phylogeographic analysis was performed by using complete PPRV genome sequence and partial N gene sequence datasets, and isolates were annotated according to their location (longitude and latitude). Partial N gene data were chosen instead of F gene data because of increased divergence reported for the N gene (*2*). For complete genome datasets, sequences from 14 viruses were considered, including 2 vaccine strains (Nigeria 1975/1 and Sungri 1996) to represent all PPRV-endemic areas. Phylogeographic diffusion along the posterior sets of trees and relationships between these locations were identified by using the Bayesian stochastic search variable selection procedure in BEAST v1.8.0 (*27*). Discrete phylogeographic analysis was performed by using the continuous time Markov chain with the flexible Bayesian skyride tree prior.

## Results

### Sequence Analysis

All 7 PPRV complete genomes are 15,948 nt and conform to the rule of 6 as described for all other morbillivirus genomes (*28*). The genome organization of the isolates was the same as that of other PPRV strains. Phylogenetic analysis of the complete genome sequences of PPRV clustered the sequences into 4 lineages. The complete genomes of PPRV isolates from Ethiopia 1994, Oman 1983, UAE 1986, and Uganda 2012 sequenced in this study belonged to lineage III and the isolates Sungri 1996, Morocco 2008, and Ethiopia 2010 belong to lineage IV. Comparison of the 12 aligned complete genome sequences showed that nucleotide differences ranged from 0.1% to 11.9%, and amino acid differences ranged from 0.1% to 7.2% (Table 2).

**Table 2:** Nucleotide and amino acid sequence differences in complete genomes of peste des petits ruminants virus lineages*

Lineage	Lineage			
	I	II	III	IV
I		5.1	6.1–7.0	5.7–6.1
II	**9.0**		5.7–6.3	4.0–4.2
III	**10.9–11–9**	**9.9–10.8**	0.2–3.0, **0.2–6.2**	6.1–7.2
IV	**10.3–10.7**	**7.2–7.6**	**10.7–11.8**	0.1–2.0, **0.1–3.2**

*Values are percentage nucleotide (bold) and amino acid sequences differences.

**Figure 1:**
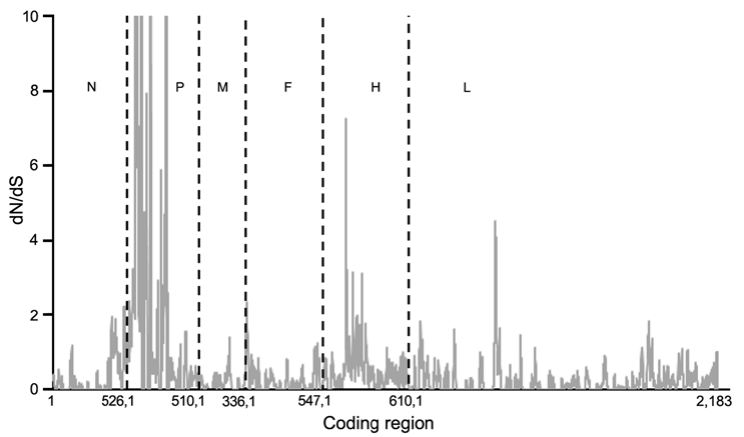
Mean ratios of nonsynonymous (dN) to synonymous (dS) substitutions per site of concatenated coding regions of peste des petits ruminants virus genome. Proportion of dS substitutions per potential dS site and proportion of dN substitutions per potential dN site were calculated by using the method of Nei and Gojobori (*29*) and the suite of nucleotide **analysis program (**www.hiv.lanl.gov). Vertical dashed lines indicate gene junctions with sliding windows of size = 5 codons. dN/dS values ≥ 10 are shown as 10. Numbers along baseline indicate coding regions (basepairs) of individual genes. N, nucleoprotein; P, phosphoprotein; M, matrix; F, fusion; H, hemagglutinin; L, large polymerase.

The dN/dS for coding regions of the various genes of PPRV (n = 12) for all 4 lineages ranged from 0.06 to 0.45 (Table 3). The dN/dS per site across the coding region of different genes of PPRV genome are shown in Figure 1. The highest dN/dS ratio was observed in the phosphoprotein gene, followed by the hemagglutinin, N, F, large polymerase, and matrix (M) genes. The relative nucleotide substitution rates at all 3 codon positions of the structural genes of PPRV showed that substitutions were more frequent at the third codon position (Table 3) as expected.

**Table 3:** Nucleotide substitution rates at codon positions of peste des petits ruminants virus genes by BEAST analysis and dN/dS by SLAC*

Gene†	Total amino acids	Codon position			Mean dN/dS
CP1.mu	CP2.mu	CP3.mu
N	526	0.44	0.33	2.23	0.13
P	510	0.81	0.69	1.49	0.45
M	336	0.48	0.15	2.36	0.06
F	547	0.46	0.26	2.29	0.10
H	610	0.57	0.37	2.06	0.19
L	2184	0.42	0.18	2.40	0.08

*BEAST, Bayesian evolutionary analysis sampling trees; dN/dS, nonsynonymous/synonymous substitutions per site; SLAC, single-likelihood ancestor counting; CP, codon position. †N = nucleoprotein; P, phosphoprotein; M, matrix; F, fusion; H, hemagglutinin; L, large polymerase.

### Evolutionary Rate Estimates

Complete genome sequences of 12 PPRV and partial N gene dataset (n = 159) were analyzed by using the coalescent-based Bayesian MCMC approach. The general time-reversible nucleotide substitution model with a gamma distribution for rate variation was selected on the basis of Akaike information criterion scores. Bayes factor test with marginal likelihood comparisons showed that the relaxed UCED clock model best fitted the PPRV complete genome and partial N gene datasets (Table 4). The 2lnBF value was >78 between UCED and strict clocks and 2–6 between UCED/uncorrelated lognormal distribution and UCED/ random clocks, which provided strong evidence for the UCED clock model. There was no difference between different demographic models compared within the UCED clock model (2lnBF <2). However, the exponential demographic model was chosen because it provided a narrow margin of 95% highest posterior density (HPD) estimates.

**Table 4:** Bayesian Markov chain Monte Carlo analysis for genomes of peste des petits ruminants virus*

Sequence dataset (no.)†	Models, substitution/ clock/demographic	Mean nucleotide substitution rate, substitutions/site/y (95% HPD)	TMRCA, y (95% HPD)	Bayes factor, –log likelihood
PPRV complete genome (12)	GTR + G/strict/BSP	3.2 x 10^–4^ (2.02 x 10^–4^–4.31 x 10^–4^)	1763 (1653–1832)	–46,972.98
	GTR + G/strict/CS	3.21 x 10^–4 ^(2.12 x 10^–4^–4.38 x 10^–4^)	1763 (1659–1834)	–46,973.06
	GTR + G/strict/EG	3.24 x 10^–4 ^(2.12 x 10^–4^–4.33 x 10^–4^)	1765 (1668–1836)	–46,973.06
	GTR + G/UCLD/BSP	2.89 x 10^-3^ (3.21 x 10^–8^–6.92 x 10^–4^)	1691 (123 bce–1944 ce)	–46,935.66
	GTR + G/UCLD/CS	3.03 x 10^–4 ^(8.99 x 10^–9^–7.07 x 10^–4^)	1705 (123–1961)	–46,935.86
	GTR + G/UCLD/EG	3.72 x 10^–4 ^(3.01 x 10^–5^–7.93 x 10^–4^)	1767 (1222–1948)	–46,935.89
	GTR + G/UCED/BSP	7.91 x 10^–4 ^(7.46 x 10^–5^–1.53 x 10^–3^)	1889 (1586–1968)	–46,933.82
	GTR + G/UCED/CS	7.98 x 10^–4 ^(8.03 x 10^–5^–1.54 x 10^–3^)	1887 (1569–1968)	–46,933.98
	**GTR + G/UCED/EG**	**9.09 x 10^–4 ^(2.13 x 10^–4^–1.64 x 10^–3^)**	**1904 (1730–1966)**	**–46,933.96**
	GTR + G/random/BSP	7.01 x 10^–4 ^(5.55 x 10^–4^–8.50 x 10^–4^)	1888 (1862–1908)	–46,934.75
	GTR + G/random/CS	6.97 x 10^–4 ^(5.38 x 10^–4^–8.41 x 10^–4^)	1887 (1860–1908)	–46,934.64
	GTR + G/random/EG	7.04 x 10^–4 ^(5.57 x 10^–4^–8.57 x 10^–4^)	1888 (1861–1908)	–46,934.89
N partial (159)	GTR + G/strict/BSP	1.22 x 10^–3^ (9.39 x 10^–4^–1.51 x 10^–3^)	1890 (1857–1917)	–2,884.524
	GTR + G/strict/CS	1.23 x 10^–3^ (9.49 x 10^–4^–1.52 x 10^–3^)	1886 (1853–1913)	–2,887.723
	GTR + G/strict/EG	1.24 x 10^–3^ (9.71 x 10^–4^–1.56 x 10^–3^)	1893 (1863–1919)	–2,885.44
	GTR + G/UCLD/BSP	1.45 x 10^–3^ (1.06 x 10^–3^–1.87 x 10^–3^)	1896 (1815–1943)	–2,806.535
	GTR + G/UCLD/CS	1.41 x 10^–3^ (1.05 x 10^–3^–1.80 x 10^–3^)	1882 (1793–1935)	–2,805.535
	GTR + G/UCLD/EG	1.49 x 10^–3^ (1.10 x 10^–3^–1.89 x 10^–3^)	1904 (1838–1943)	–2,805.921
	GTR + G/UCED/BSP	1.52 x 10^–3^ (1.11 x 10^–3^–1.98 x 10^–3^)	1904 (1817–1949)	–2,799.572
	GTR + G/UCED/CS	1.46 x 10^–3^ (1.05 x 10^–3^–1.88 x 10^–3^)	1886 (1785–1940)	–2,799.512
	**GTR + G/UCED/EG**	**1.56 x 10^-3^ (1.16 x 10^–3^–1.99 x 10^–3^)**	**1910 (1846–1947)**	**–2,799.444**
	GTR + G/random/BSP	1.26 x 10^–3^ (9.44 x 10^–4^–1.58 x 10^–3^)	1881 (1837–1915)	–2,865.846
	GTR + G/random/CS	1.24 x 10^–3^ (9.38 x 10^–4^–1.57 x 10^–3^)	1875 (1831–1910)	–2,866.111
	GTR + G/random/EG	1.27 x 10^–3^ (9.62 x 10^–4^–1.60 x 10^–3^)	1880 (1841–1914)	–2,866.929
N CDS (12)	GTR + G/UCED/EG	1.01 x 10^–3^ (2.79 x 10^–4^–1.83 x 10^–3^)	1924 (1799–1970)	NA
N complete gene (12)	GTR + G/UCED/EG	1.08 x 10^–3^ (3.19 x 10^–4^–1.93 x 10^–3^)	1923 (1804–1970)	NA
P CDS (12)	GTR + I/UCED/EG	1.11 x 10^–3^ (3.46 x 10^–4^–1.29 x 10^–3^)	1931 (1833–1972)	NA
P complete gene (12)	GTR + I/UCED/EG	1.19 x 10^–3^ (3.46 x 10^–4^–2.03 x 10^–3^)	1930 (1828–1971)	NA
M CDS (12)	GTR + G/UCED/EG	6.52 x 10^–4 ^(1.20 x 10^–4^–1.20 x 10^–3^)	1897 (1695–1964)	NA
M complete gene (12)	GTR + I/UCED/EG	2.49 x 10^–3^ (9.96 x 10^–4^–4.14 x 10^–3^)	1944 (1879–1973)	NA
FCDS (12)	GTR + I/CED/EG	8.95 x 10^–4 ^(2.43 x 10^–4^–1.58 x 10^–3^)	1914 (1766–1968)	NA
F complete gene (12)	GTR + G/UCED/EG	1.33 x 10^–3^ (3.26 x 10^–4^–2.36 x 10^–3^)	1912 (1754–1967)	NA
H CDS (12)	GTR + G/UCED/EG	1.21 x 10^–3^ (3.96 x 10^–4^–2.04 x 10^–3^)	1926 (1826–1969)	NA
H complete gene (12)	GTR + G/UCED/EG	1.25 x 10^–3^ (4.34 x 10^–4^–2.14 x 10^–3^)	1925 (1821–1968)	NA
L CDS (12)	GTR + I/UCED/EG	9.82 x 10^–4 ^(3.76 x 10^–4^–1.67 x 10^–3^)	1929 (1834–1969)	NA
L complete gene (12)	GTR + I/UCED/EG	9.69 x 10^–4 ^(3.36 x 10^–4^–1.64 x 10^–3^)	1927 (1820–1969)	NA
PPRV/RPV/MV (16)	GTR+ G + I/UCED/EG	1.89 x 10^–3^ (5.55 x 10^–4^–3.31 x 10^–3^)	1616 (1072–1859)	NA

*Bold indicates best-fit models. HPD, highest posterior density; TMRCA, time to most recent common ancestor; GTR + G, general time-reversible with gamma distribution rates; BSP, Bayesian skyline plot; CS, constant size; EG, exponential growth; UCLD, uncorrelated lognormal distribution; UCED, uncorrelated exponential distribution; NA, not applicable; GTR + I, general time-reversible with invariant sites; GTR+ G + I, general time-reversible with gamma distribution rates and invariant sites. †PPRV, peste des petits ruminants virus; N, nucleoprotein; CDS, coding sequence; P, phosphoprotein; M, matrix; F, fusion; H, hemagglutinin; L, large polymerase; RPV, rinderpest virus; MV, measles virus.

Accordingly, the UCED and exponential growth model have been directly used for the individual PPRV gene dataset and the PPRV/RPV/MV complete genome dataset to estimate the TMRCA and substitution rate per site per year. When we used the UCED and exponential growth models, we found that the mean evolutionary substitution rate of the PPRV complete genome was estimated to be 9.09 × 10^−4^ (95% HPD 2.13 × 10^−4^–1.64 ×10^−3^). When 2 complete genome sequences of vaccine strains were added into this analysis, the same models (general time-reversible nucleotide substitution model with a gamma distribution, UCED, and the exponential growth demographic models) were best fitted, and the mean substitution rate/site/year was reduced to 7.86 × 10^−4^ (95% HPD 2.17 × 10^−4^–1.4 × 10^−3^). Furthermore, the evolutionary nucleotide substitution rate for combined PPRV/RPV/MV complete genomes was 1.89 × 10^−3^ (95% HPD 5.55 × 10^−4^–3.31 × 10^−3^). Analysis of individual genes of the PPRV coding region dataset, coding and noncoding region datasets, and partial N gene dataset are shown in Table 4.

### Temporal Dynamics

**Figure 2:**
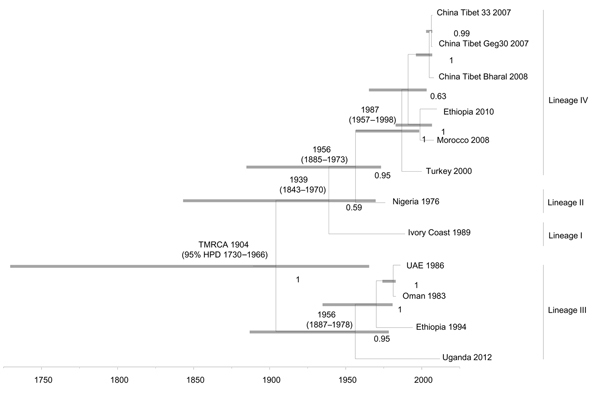
Time-scaled Bayesian maximum clade credibility phylogeny tree based on peste des petits ruminants virus complete genome sequences. The tree was constructed by using the uncorrelated exponential distribution model and exponential tree prior. Branch tips correspond to date of collection and branch lengths reflect elapsed time. Tree nodes were annotated with posterior probability values and estimated median dates of time to most recent common ancestor (TMRCA). Corresponding 95% highest posterior density (HPD) interval values of TMRCA are indicated as gray bars. Horizontal axis indicates time in years. UAE, United Arab Emirates.

A Bayesian time-scaled MCC tree based on complete PPRV genomes was constructed (Figure 2) by using the UCED model with exponential growth demography. The estimated median TMRCA of PPRV for all 4 lineages and divergence of lineage III PPRV were found to be ≈1904 (95% HPD 1730–1966). Lineage I diverged in ≈1939 (95% HPD 1843–1970). Lineages II and IV diverged from each other in ≈1956 (95% HPD 1885–1973). The TMRCA for lineage III viruses (n = 4) used in this study was estimated to be ≈1956 (95% HPD 1887–1978). TMRCA for lineages I and II PPRV were not predicted because only 1 virus from each lineage was used. The TMRCA for lineage IV viruses (n = 6) used in this study was estimated to be ≈1987 (95% HPD 1957–1998). When both Nigeria 1975/1 and Sungri 1996 vaccine strains were included in the study, the TMRCA for all lineages of PPRV shifted from 1904 (95% HPD 1730–1966) to 1891 (95% HPD 1705–1960). Analysis of the partial N gene dataset showed the TMRCA as 1910 (95% HPD 1846–1947) for all lineages of PPRV, 1960 (95% HPD 1941–1971) for lineage III, 1958 (95% HPD 1946–1971) for lineage I, 1961 (95% HPD 1941–1967) for lineage II, and 1987 (95% HPD 1969–1988) for lineage IV.

**Figure 3:**
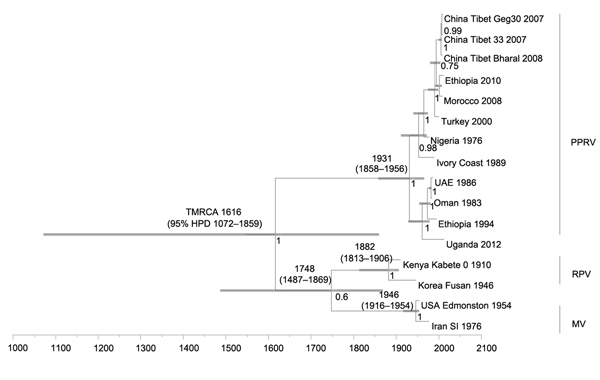
Time-scaled Bayesian MCC phylogeny tree based on peste des petits ruminants virus (PPRV), rinderpest virus (RPV), and measles virus (MV) complete genome sequences. The tree was constructed by using the uncorrelated exponential distribution model and exponential tree prior. Branch tips correspond to date of collection and branch lengths reflect elapsed time. Tree nodes were annotated with posterior probability values, estimated median dates of time to most recent common ancestor (TMRCA). Corresponding 95% highest posterior density (HPD) values of TMRCA are indicated as gray bars. Horizontal axis indicates time in years. UAE, United Arab Emirates.

Results of TMRCA analysis using complete coding and coding and noncoding regions of individual PPRV genes are shown in Table 4. If one considers coding and noncoding sequences of individual genes in the analysis, a difference in TMRCA was found only for the M gene (i.e., 1944, 95% HPD 1879–1973). The TMRCA of PPRV/RPV/MV was estimated to be ≈1616 (95% HPD 1072–1859), and the TMRCA for PPRV was estimated to be 1931 (95% HPD 1858–1956) (Figure 3).

### Population Demography of PPRV

**Figure 4:**
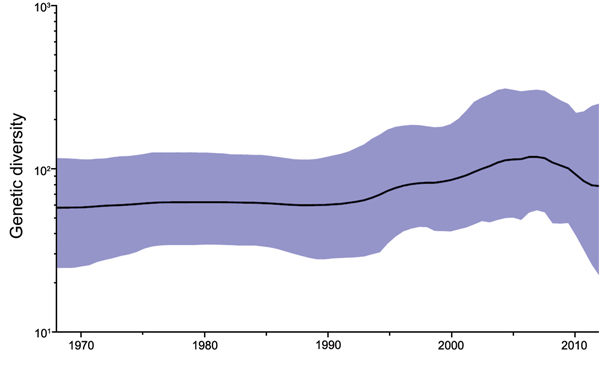
Bayesian skyline plot showing demographic history of global peste des petits ruminants viruses sampled during 1968–2012. Genetic diversity was estimated by using a partial nucleoprotein gene dataset (n = 159). The thick black line represents median genetic diversity and the blue shaded areas show 95% highest posterior density estimate.

The demographic history of PPRV was investigated by using the partial N gene sequence dataset according to the BSP method implemented in BEAST. The BSP with an assumed piecewise-constant model has facilitated estimation of effective population size through time. The BSP showed that the population did not show much genetic diversity (effective number of infections) until the mid-1990s when diversity started to increase. Toward the first decade of the 21st century, the population size appeared to reach a peak and then showed a small decrease until the most recent sampling in 2012 (Figure 4). The HPD interval size for the plot is narrow, which indicates strong support for this population trend.

### Phylogeographic Analysis

**Figure 5:**
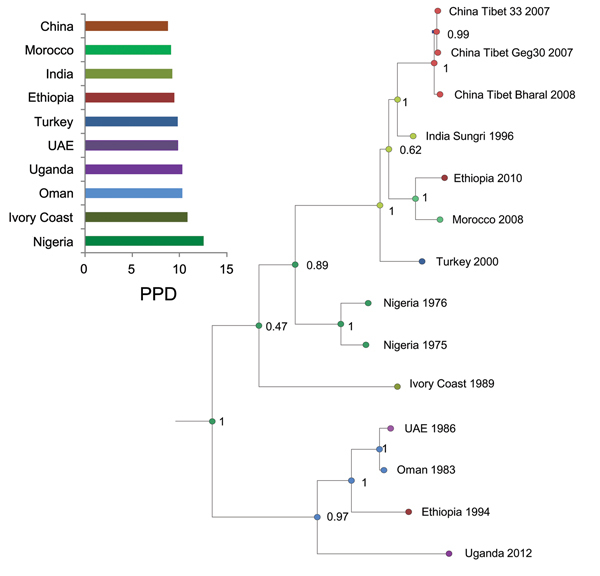
Maximum clade credibility tree constructed for the geospatial analysis of peste des petits ruminants viruses by using complete genome data. Nodes are colored according to the most probable location of their ascendent locations. Posterior probability values are shown along tree nodes. Posterior probability distribution (PPD) values of root location states of the ancestral node are shown along the x-axis at the top left. UAE, United Arab Emirates.

To estimate the geographic origin of PPRV, we summarized the results of Bayesian phylogeographic analyses by visualizing the annotated MCC tree (Figure 5). The complete genome sequence data used in this analysis incorporated all 14 isolates, including the vaccine strains, from 10 discrete locations so as not to leave out any reported virus-endemic area. The root state posterior probabilities for all the locations ranged between 9.02% and 12.69%; Nigeria and the Ivory Coast (now Côte d’Ivoire) receiving marginally higher support, 12.69% and 10.53%, respectively, than the rest of the locations (Figure 5).

**Figure 6:**
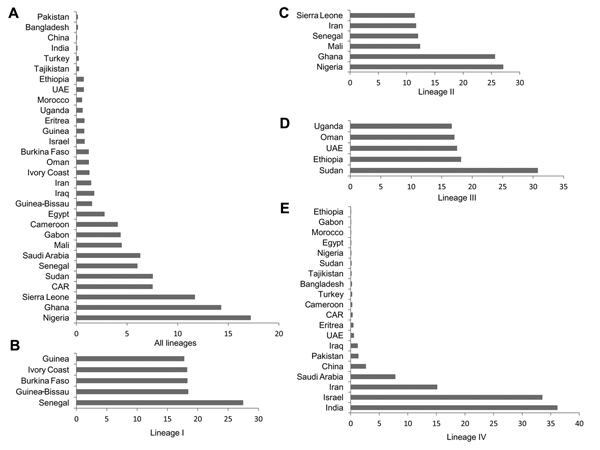
Probability of root locations of the most recent common ancestral peste des petits ruminants (PPRV). MCC trees were obtained by using the continuous time Markov chain and Bayesian stochastic search variable selection procedures. Root location probabilities of the most recent common ancestor using global PPRV isolates (panel A ) are shown graphically alongside lineages I–IV (panels B–E) and were estimated by using a complete dataset of PPRV partial nucleoprotein gene data and individual lineages separately. Probabilities of root locations are shown as percentages along the x-axes. UAE, United Arab Emirates; CAR, Central African Republic.

Because the geographic origin of PPRV could not be localized to a single country by using 14 complete genome sequences, further phylogeographic analysis was performed by using 159 partial N gene sequences collected from 30 locations during 1968–2012. The root state posterior probabilities of PPRV ranged from 0.11% to 17.20%, and Nigeria (17.20%), Ghana (14.28%), and Sierra Leone (11.68%) showed the highest marginal support (Figure 6). The highest marginal support of root state posterior probabilities indicated that the geographic origin of lineage I PPRV was Senegal (27.44%), that of lineage II PPRV was Nigeria (27.00%), that of lineage III PPRV was Sudan (30.73%), and that of lineage IV PPRV was India (36.00%).

## Discussion

We sequenced complete genomes of 4 lineage III and 3 lineage IV isolates of PPRV. We used these genomes and other available genomes to assess the evolutionary substitution rate, TMRCA, and divergence of PPRV lineages and the geographic origin of PPRV.

The measure of selective pressures acting across the PPRV genome showed only purifying (stabilizing) selection occurring across the genome and no evidence of positive selection. The conservation of amino acid residues was further confirmed by the fact that the relative substitution rates at the third codon position of all the genes were higher than those for the first and second codon positions. The observed upper limit of 11.9% nt divergence (7.2% aa divergence) among PPRVs is consistent with the low level of antigenic divergence observed because despite lineage differentiation, only a single serotype exists for PPRV. Homologous recombination events are generally rare or absent in negative-sense RNA viruses (*30*) and thus could not have been evaluated in this study.

From a genetic perspective, substitution rates are critical parameters for understanding virus evolution, given that restrictions in genetic variation within a population of viruses can lead to lower adaptability and pathogenicity (*31*). Our analyses estimated a range of PPRV nucleotide substitution rates throughout the complete genome of 1.64 × 10^−3^–2.13 × 10^−4^ substitutions/site/year, which is similar to that predicted for other paramyxoviruses (10^−3^–10^−4^ substitutions/site/year) (*32**–**35*). Despite low levels of antigenic divergence, as shown by existence of a single serotype, genome plasticity of PPRV might explain its ability to emerge and adapt in new geographic regions and hosts, as reported extensively across vast areas in recent years. The TMRCA of PPRV obtained from complete genome sequence was estimated to be during 1904 (95% HPD 1730–1966). Similarly, the estimated TMRCA obtained from individual gene sequence, partial N gene sequence of PPRV, and combined PPRV/RPV/MV complete genome sequences was during 1910–1944.

That the predicted TMRCA for PPRV was during the early 20th century is reasonable because the first recorded description of PPRV was made in 1942 (*36*). The delay of a few decades before identification of PPRV as a distinct viral entity after its initial detection can likely be attributed to confusion in differentiation between PPRV and RPV, a virus for which extensive cross-neutralization is observed after vaccination and natural infection, and lack of differentiating diagnostic tools. Substitution rates were consistent across each gene for PPRV. However, greater substitution rates were observed in the GC rich regions of the F and M genes. Similarly, the substitution rate was greater, as predicted because of the variability seen at the nucleotide level, in the highly variable region of the N gene sequence (255 nt) for PPRV. The TMRCA estimation was not possible for lineage I and II viruses (vaccine strain was omitted) because only 1 complete genome sequence was available for each lineage. Therefore, more complete genome sequences are required to study evolutionary and phylogenetic relationships for these lineages.

Biased estimates in substitution rate and TMRCA were observed by using datasets that included tissue culture–passaged, attenuated vaccine strain complete genome sequences, in which slower evolutionary substitution rates and earlier TMRCA were predicted. Similar observations were reported for PPRV/RPV/MV N gene sequence analyses, in which a slower and biased nucleotide substitution rate was observed when vaccine strain sequences (*33*) were included in the analysis and faster substitution rates and later TMRCA predictions were suggested when vaccine strain sequence data were excluded (*34*).

Spatial and temporal dynamics of RNA viruses are often reflected by their phylogenetic structure (*37*). Potential divergence events for different PPRV lineages were inferred by using rooted, time-measured phylogenetic trees with higher confidence from the PPRV complete genome sequence dataset. The inferred phylogeny supports the initial divergence of lineage III isolates, followed by lineage I isolates; lineage II and IV isolates were predicted to have diverged from each other at a later time. The inference of divergence events presented facilitated a better understanding of historical divergence of PPRV and offered further opportunities to study viral demographic history and dispersal events.

The demographic analysis of PPRV with the BSP indicated historically constant genetic variability of PPRV over time. This finding could be a reflection of the use of RPV vaccine in small ruminants to protect animals against PPRV through the 1990s, which might have affected the evolution and spread of PPRV. In the early 21st century, genetic diversity of PPRV has gradually increased, which reflects frequent outbreak reports. The increased genetic diversity may be a driver for selection pressures within individual lineages and might result in extinction events, as suggested by an absence of lineage I virus. In recent years, as efforts have increased to actively control and eradicate PPRV, a decrease in genetic diversity has been observed.

Phylogeographic reconstruction with spatial and temporal information of virus isolates has enabled an understanding of the historic emergence and dispersal patterns involved in virus evolution (*38*). Although PPRV existed earlier than its first description in Ivory Coast in 1942 (*39*), PPRV was later reported in Senegal, Chad, Togo, Benin, Ghana, Nigeria, Oman, Sudan, Saudi Arabia, India, Jordan, Israel, Ethiopia, Kenya, Uganda, and Pakistan (*40*). Our phylogeographic analysis indicated that Nigeria was the geographic origin of the most recent common ancestor of PPRV because of the highest root location state probability. Furthermore, geographic origins of the most recent common ancestor of PPRV lineages I, II, and III were predicted to be across Africa; lineage IV likely emerged in India. In conclusion, these findings suggest that the origin of PPRV was in western Africa, which then spread to eastern Africa, the Middle East, and Asia. However, although these predictions are suggestive of a potential origin for PPRV, caution must be exercised in their interpretation because estimates of geographic origin rely on available datasets, and these datasets need enhancing to provide greater confidence for phylogenetic assessment. As more sequence data become available for PPRV and the other morbilliviruses, ancestral origins of each virus and intraspecies differentiation might become more clear.
